# Economic evaluations of early detection strategies for pancreatic cancer: a systematic review

**DOI:** 10.1007/s10198-025-01793-4

**Published:** 2025-06-05

**Authors:** Robert Wittram, Léon Kreis, Hans-Helmut König, Christian Brettschneider

**Affiliations:** Department of Health Economics and Health Services Research, Hamburg Center for Health Economics, University Medical Center Hamburg-Eppendorf, Robert Wittram, Martinistr. 52/ Gebäude West 37, D-20246 Hamburg, Germany

**Keywords:** Pancreatic cancer, Early detection, Economic evaluation, Systematic review

## Abstract

**Objectives:**

The early detection of pancreatic cancer is an important step in reducing mortality by offering patients curative treatment. The aim of this study was to synthesize available evidence on the costs and cost-effectiveness of strategies for early pancreatic cancer detection.

**Methods:**

The electronic databases PubMed, Web of Science, and EconLit were searched for peer-reviewed and published papers in English until April 2024 with no date or contextual restrictions. Economic evaluations of early pancreatic cancer detection strategies compared to alternative or no detection strategies were criteria for inclusion.

**Results:**

Thirty-one articles were included, 22 were full and nine were partial economic evaluations. Fifteen studies screened target populations with pancreatic cancer-associated risk factors and 16 conducted surveillance of patients with precancerous lesions. Six studies found early detection strategies to be cost-effective, one did not, and thirteen reported partially cost-effective results. In all studies, populations of interest had an elevated pancreatic cancer risk compared to the general population. Endoscopic ultrasound, magnetic resonance imaging, and computed tomography were the most frequently evaluated imaging modalities. Patient engagement, valuation of outcomes and choice of discount rates were among incomplete reporting categories, and narrow evaluation perspectives may have biased the results.

**Conclusions:**

Early detection strategies for pancreatic cancer may be cost-effective for certain high-risk patient groups. However, evaluations so far have applied heterogeneous methods, used different modalities, had various target groups and screened at different frequencies. Further evaluations will be required to systematically synthesize economic evidence regarding specific early detection strategies.

**Registration:**

PROSPERO registration CRD42023475348.

**Supplementary Information:**

The online version contains supplementary material available at 10.1007/s10198-025-01793-4.

## Introduction

In 2022, pancreatic cancer was diagnosed in 510,000 individuals globally. In the same year, 467,000 died of it [1]. In Europe, the disease is likely to establish itself as one of the top three causes of cancer death [2]. The 5-year survival rate for pancreatic cancer has improved over the past decades, but this improvement has only been modest compared to other cancer types [3]. The risk of pancreatic cancer increases with age and the ability to diagnose the disease has improved over time, which explains the increase in pancreatic cancer incidence especially in high-income nations [4]. Alongside this epidemiological trend, pancreatic cancer care will increasingly demand resources in the future. Particularly in high-income countries, an increasing disease burden due to demographic change can be expected [5]. A Swedish study predicts a rise in total costs from €125 million in 2018 to €225 million in 2030 for pancreatic cancer [6]. Pancreatic cancer is thus gaining economic relevance.

While survival benefits have been achieved for patients undergoing resection, this has not been achieved for tumours that cannot be surgically resected [7]. This highlights the importance of early detection to achieve survival gains. Screening strategies in subgroups at increased risk of pancreatic cancer may enable more patients to receive life-prolonging treatment [8]. Understanding economic aspects of early detection is necessary to guide decision-making in health care systems. The allocation of more resources towards secondary prevention of pancreatic cancer alongside improvements in curative treatment may support reducing the overall mortality and economic burden.

Current early detection modalities for pancreatic cancer include imaging and blood tests. In the asymptomatic course of early pancreatic cancer, a combination of imaging methods is often applied. The American Gastroenterological Association recommends a combination of magnetic resonance imaging (MRI) and endoscopic ultrasonography (EUS) to detect pancreatic neoplasms at resectable stages [9]. Computed tomography (CT) is acknowledged as a widely available and rapid method to scan for pancreatic neoplasms [10]. Artificial intelligence-supported imaging and blood sample-based methods are promising technological advances in the early detection of pancreatic cancer [11].

As the prevalence of pancreatic cancer is relatively low and due to technologically limited modalities at current times, screening high numbers of standard-risk patients would lead to high costs per detected case and an ethically unacceptable number of false-positive patients [12]. Thus, guidelines recommend screening of individuals who have an elevated pancreatic cancer risk [13]. The elevated risk in subgroups (e.g. specific gene mutation carriers) may result in screening to be cost-effective.

Pancreatic cancer patients have a short median survival of 10–12 months [3]. Less than 20% of all patients are surgically resectable, so the majority of patients, that cannot be curatively treated, rely on palliative care for a short time period [14]. The health-related quality of life (HRQoL) of these patients at advanced cancer stages is relatively low and evidence on whether HRQoL can be improved is mixed [15]. Early detection can ideally increase the proportion of resectable patients, which prolongs survival and thus increases the long-term relevance of HRQoL. This long term relevance of HRQoL in resected patients was noted in a systematic review [16]. HRQoL in pancreatic cancer patients can be measured using generic scales such as the 5-dimension EuroQol questionnaire (EQ-5D). To better understand the benefits of pancreatic cancer screening, it is important to understand how HRQoL has been included in economic evaluations to date alongside changes in survival.

This study only focuses on economic aspects of early detection strategies as part of pancreatic cancer secondary prevention. This may support resource allocation towards early detection by synthesizing cost-effective strategies. This review will rely on findings from health economic evaluations. Full economic evaluations systematically compare costs and effects of at least two interventions [17]. Partial economic evaluations, which are limited to comparison of costs, will also be considered due to the first-time conduct of this review. A previous systematic review analysed economic evaluations up until 2015 covering all aspects of pancreatic cancer care [18]. This review, which focuses specifically on early detection of pancreatic cancer and its economic outcomes has not been conducted. The systematic comparison of the studies with respect to study quality, types of screening strategies and conclusions on economic feasibility will inform future research.

## Methods

The systematic review was developed in accordance with the Preferred Reporting Items for Systematic Review and Meta-analyses (PRISMA) statement [19].

### Search strategy

A formal screening of PubMed, Web of Science, and EconLit was conducted. The search was run from 01 November, 2023 to 25 April, 2024. Studies had to be written in English and needed to be published in peer-reviewed journals. The search terms that were used for the respective databases can be found in Appendix 1. The search strategy was developed in collaboration with a librarian. Search terms targeting economic evidence were previously validated for searching economic evaluations in PubMed [20]. The disease specific search terms were adapted from a previous systematic review [18]. The references of previous systematic reviews on adjacent topics were hand-searched to identify articles of potential interest and a simple search in the CEA registry was run. The study protocol was registered at the International prospective register of systematic reviews (PROSPERO no. CRD42023475348) and published in a peer-reviewed journal [21].

### Inclusion criteria

Studies that met all of the following criteria were included in the systematic review:

- *Participants*: adult patients at risk of pancreatic cancer or its subtypes.
- *Intervention(s)*: strategies with the aim of detecting pancreatic cancer early (i.e. screening, surveillance, diagnosis and early detection strategies).
- *Comparator(s)*: the standard of care (i.e. no early detection) or alternative detection strategies.
- *Outcomes*: costs and cost-effectiveness of early detection.
- *Context*: no application of geographic, ethnic, time or sociocultural restrictions.
- *Types of studies*: full economic evaluations comparing at least two strategies based on costs and effects; partial economic evaluations only analysing costs were also eligible.

Studies with no full-text article available were not selected. Non-original research (e.g. editorials or conference abstracts), systematic reviews, meta-analyses, and qualitative designs were excluded.

### Study selection

The search results were merged into one file and managed using Endnote 21 (The Endnote team, Clarivate, Philadelphia, PA, USA) and duplicates were removed. RW and LK independently performed title and abstract screening. Any discrepancies were discussed and resolved by consensus. A third reviewer (CB) was consulted in cases of uncertainty. After retrieval of all full texts, a second round of screening was performed independently among the eligible first round citations. Reasons for exclusion were documented and discrepancies were resolved by consensus between the reviewers. The literature selection process was documented and visualized in a PRISMA flow diagram [19].

### Data extraction

Data was extracted from the included literature into three data tables. First, the study type and its analytic approach were determined alongside economic evaluation-specific information. Additionally, the screened population(s) and the general early detection approach were displayed. Second, the study input parameters were collected in more detail including start age for early detection, the frequency of testing. Clinical information on pancreatic cancer type and assumed risk were furthermore included. The input costs were extracted as raw numbers, currency and year for the most commonly used modalities. Further costs were listed as categories and the type of sensitivity analysis to be performed was shown. Third, the main study outcomes were included as categories. Incremental cost-utility ratios were displayed as raw numbers and the assumed cost-effectiveness threshold. Based on the two parameters, each cost-utility analysis was labelled not, partly or indeed cost-effective. Partly cost-effective strategies had uncertain results around the assumed threshold. Additional information on the utility input parameters that were used by respective studies was collected as a fourth domain.

### Quality assessment and risk of bias

The quality assessment was conducted independently by RW and LK. Any disagreements were resolved by consensus. The completeness of reporting was assessed following the Consolidated Health Economic Evaluation Reporting Standards (CHEERS) statement [22]. This list was found to be suitable for methodologically heterogeneous study samples. Its criteria were scored as reported, not reported or not applicable. The completeness of the respective items across all studies was analysed. The presence of an item in less than 25% of all studies was judged as “incomplete”, in more than 75% as “complete” and in the quartiles in between as “mixed”. Sources of bias in the reviewed economic evaluations were identified using the Bias in Economic Evaluation (ECOBIAS) checklist, which contains 22 biases that can may occur in economic evaluations [23]. The biases were individually scored as “yes”, “no”, “unclear” or “not applicable”. When in more than half of all studies a bias was present, the risk of bias was categorized as “present”. When present in 10–50%, risk of bias was “likely present” and “not present” when below 10%.

### Patient and public involvement

Patients or the public were not involved in the design, or conduct, or reporting, or dissemination plans of this research project.

## Results

**Fig. 1:**
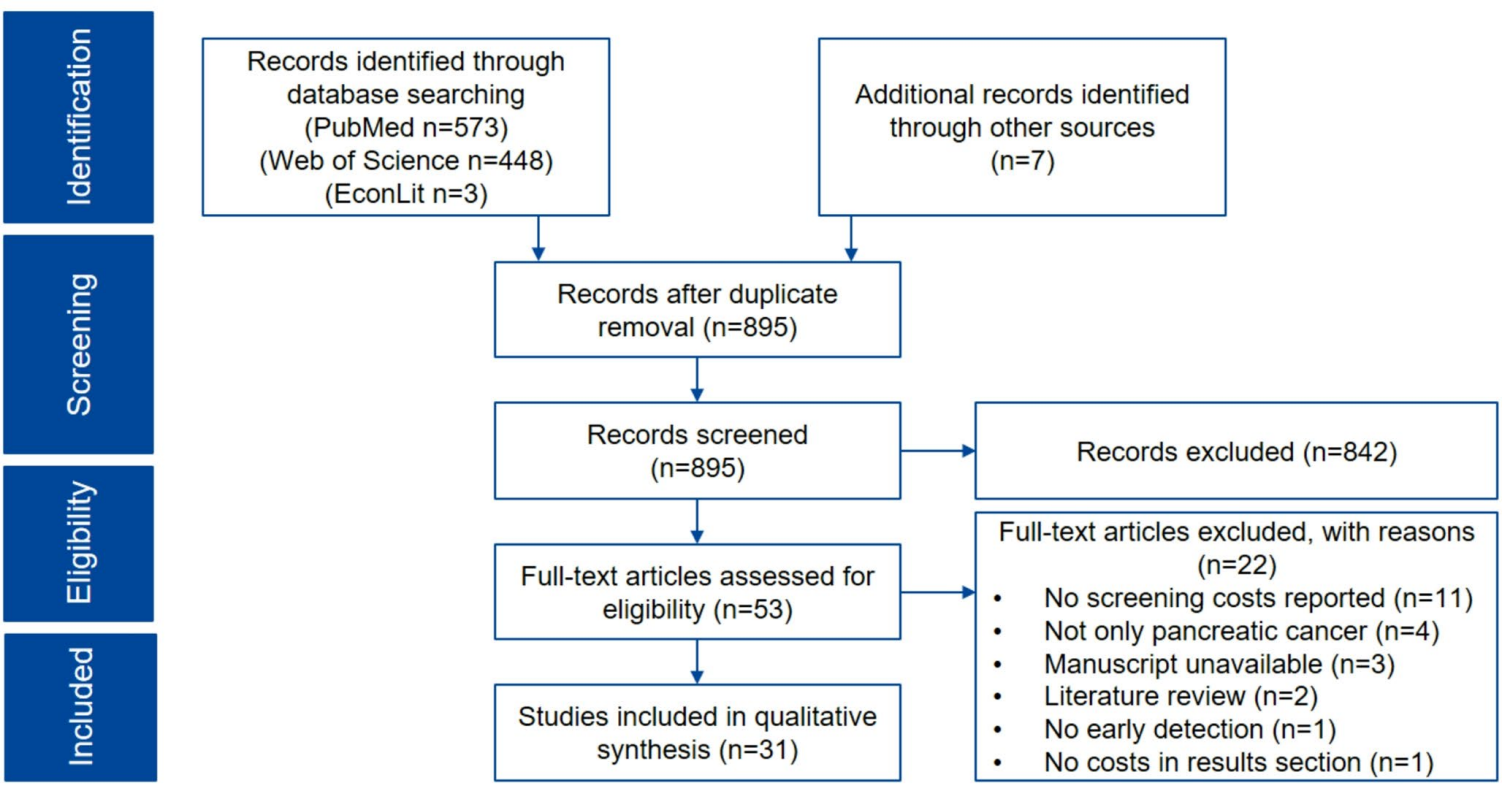
PRISMA flowchart diagram for illustration of the literature selection process

Figure 1 illustrates the search process for economic literature as a PRISMA flowchart diagram. The database search yielded 573, 448 and 3 hits in PubMed, Web of Science and EconLit, respectively. Seven additional studies were identified by searching the CEA registry and via hand searching the references of included studies. Out of these in total 1031 hits, 136 duplicates were removed. 895 titles and abstracts were screened for eligibility. 53 studies were subsequently full-text screened. No reporting of screening costs in the study output section (*n* = 11), focusing on other cancer types besides pancreatic cancer (*n* = 4) and unavailable manuscript (*n* = 3) were the most frequent reasons for exclusion. The formal literature screening resulted in 31 original papers that were included [12, 24–53].

### Quality assessment and risk of bias

**Fig. 2:**
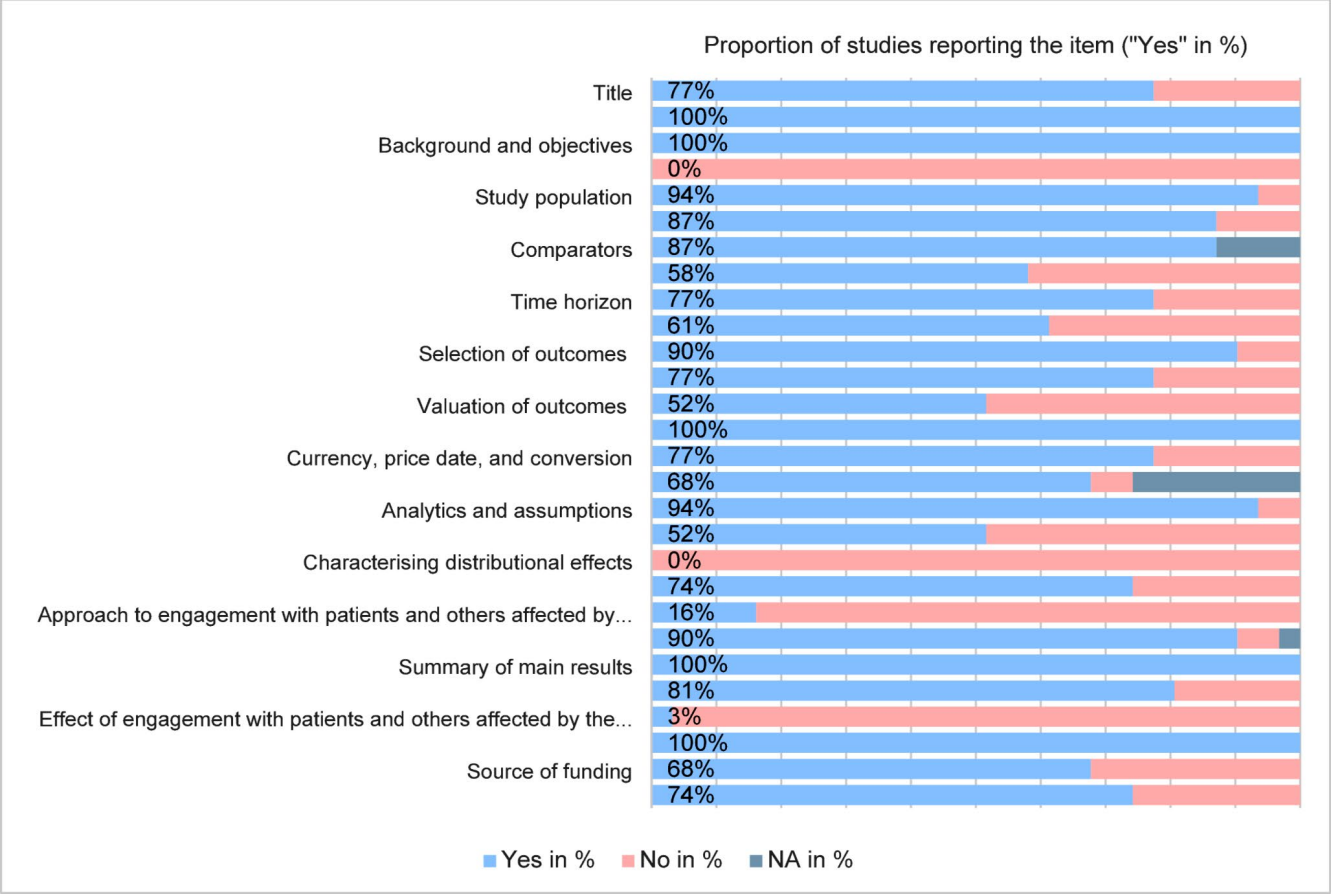
Completeness of reporting results according to 28 CHEERS checklist items

The reporting quality of the 31 included studies was assessed using the CHEERS checklist 2022 [22] and the results are shown in Fig. 2. Out of 28 items, 16 items were categorized as complete and four items as incomplete. The latter included the engagement of patients in the study design, characterising distributional effects and presenting a health economic analysis plan. Eight items were located in the two quartiles between, so the completeness was mixed. This included model-specific items such as study perspective, discount rate, outcome valuation and model rationale. Furthermore, general items such as funding source and conflict of interest showed mixed results. Risks of bias were identified using the ECOBIAS checklist. The results are shown in Appendix 2. Narrow perspective, reporting and dissemination, and internal consistency-related bias were found to be the most present risks of bias. Moreover, inappropriate discounting and quality-of-life weights were frequently present in the study sample. The last eleven items were model-specific and thus only applicable to a limited extent.

### Study characteristics and analytic frameworks

The majority of all studies were published in 2015 and onwards (*n* = 23/31; 74%) (Table 1). Most studies originated in the United States (*n* = 16/31; 52%), the second most in Sweden (*n* = 4/31; 13%), and the remaining studies were from other countries (*n* = 11/31; 35%). Publications were either classified as full (*n* = 22/31; 71%) or partial economic evaluations (*n* = 9/31; 29%). Full economic evaluations were either cost-utility (*n* = 20/22; 77%) or cost-effectiveness analyses (*n* = 2/22; 9%). The most common analytic approaches (*n* = 15/22; 68%) were Markov models and decision tree designs (*n* = 6/22; 27%). A full trial-based evaluation was conducted by two studies. An individual-level simulation was performed in one study. Partial economic evaluations (*n* = 9/31; 29%) were subdivided into studies that calculated early detection costs based on primary data (*n* = 6/9; 67%) or in hypothetical patient cohorts (*n* = 3/9; 33%).

**Table 1:** Summary of analytic framework and early detection strategy

Author(s), year, country	Study type	Analytics	Perspective	Time horizon	Discount rate	Screened population(s)	Early detection strategies
**Screening**							
Bruenderman and Martin, USA [27]	Cost-Analysis	DT model	NAv	3 and 20 years	NAv	HRI: PJS, HP, FPC, p16-Leiden mutations, NOD	MRI/MRCP and CA19-9
Corral et al., USA [30]	CUA	Markov model	Third-party payer	Lifetime	3%	HRI: FPC, PJS, HP, Lynch syndrome, 5 gene mutations	No screening vs. EUS vs. MRI
Draus et al., Sweden [12]	CUA	NAv	Societal	30 and 40 years	3%	Hypertension, daily smoker, FPC, HP, NOD, early symptoms	Hypothetical biomarker test: pre- vs. post-screening outcomes
Ghatnekar et al., Sweden [34]	CUA	Markov model	Societal	Lifetime	NAv	NOD patients	Hypothetical biomarker test vs. no screening
Ibrahim et al., The Netherlands [37]	CUA	Trial & Markov model	Healthcare sector	Lifetime	4% (C) 1.5% (E)	Patients with a CDKN2A-p16‐Leiden founder mutation	MRI (+ EUS) vs. no screening
Joergensen et al., Denmark [38]	CUA	Trial-based evaluation	NAv	5 years	4%	Patients with HP or suspected FPC	EUS (+ CA19-9) vs. no screening
Kowada, Japan [39]	CUA	Markov model	Healthcare sector	Lifetime	3%	FPC patients	Screening (US, MRI, EUS, CT or PET) vs. no screening
Kowada, Japan [40]	CUA	Markov model	Third-party payer	Lifetime	3%	Type 2 diabetes; NOD; diabetes with detected pancreatic lesion	US, MRI, CA 19 − 9, EUS, CT, microRNA or PET vs. no screening
Kumar et al., USA [41]	CUA	DT model	Third-party payer	Lifetime	3%	FPC, PJS, FAMM, HP, BRCA2	EUS vs. no screening
Peters et al., USA [45]	CUA	Microsimulation model	Healthcare sector	Lifetime	3%	Eight germline pathologic variants	MRI (+ EUS) vs. no screening
Rubenstein et al., USA [48]	CUA	Markov model	Third-party payer	Lifetime	3%	Men from FPC kindreds and findings of chronic pancreatitis	Surgery vs. EUS vs. EUS and FNA vs. no screening
Rulyak et al., USA [49]	CEA	DT model	Third-party payer; Societal	Lifetime	3%	Members of FPC kindreds	EUS vs. no screening
Schwartz et al., USA [50]	CUA	Markov model	Healthcare sector	Lifetime	3%	NOD patients and END-PAC score > 0 (PC risk predictor)	One-time CT vs. no screening
Wang et al., USA [52]	CUA	Markov model	Healthcare sector	Lifetime	3%	NOD patients	MRI/ MRCP/ EUS/ FNA vs. no screening
Zubarik et al., USA [53]	Cost analysis	Trial-based evaluation	NAv	NAv	NAv	Patients with suspected FPC	CA 19 − 9 (EUS to confirm) vs. standard
**Surveillance**							
Alvarez et al., USA [24]	Cost analysis	Trial-based evaluation	NAv	NAv	NAv	Patients diagnosed with PC	2 Noninvasive and 10 Invasive diagnostic tests
Aronsson et al., Sweden [25]	CUA	Markov model	NAv	35 years	3%	Patient with an incidentally found low-risk BD-IPMN	Surgery vs. EUS & MRI vs. Watch & Wait
Bosch et al., Spain [26]	Cost analysis	Trial-based evaluation	NAv	NAv	NAv	Patients with PC diagnosis hospital admission	Ambulatory quick-diagnosis-unit vs. Inpatient setting
Chen et al., USA [28]	Cost analysis	DT model	Third-party payer	NAv	NAv	Patients with detected pancreatic mass lesions	CT-/US-FNA vs. ERCP-B vs. EUS-guided FNA vs. Biopsy
Chen et al., Canada [29]	Cost analysis	Trial-based evaluation	Healthcare sector	NAv	NAv	Patients with a pancreatic mass on CT or MRI	EUS and fork-tip needle biopsy vs. EUS-FNA
Das et al., USA [31]	CUA	Markov model	Third-party payer	Lifetime	3%	Patients with a pancreatic cyst neoplasm	Wait & Watch vs. Surgery vs. Risk-stratify vs. Risk-stratify + panel
Faccioli et al., Italy [32]	CUA	Markov model	Healthcare sector	10 years	NAv	Patients with pancreatic cysts	CEUS follow-up vs. guidelines
Ghaneh et al., UK [33]	CUA	Trial-based & DT model	Health care	1 year	NAv	Patients with pancreatic cyst, jaundice or elevated CA19-9 level	PET + CT vs. Multidetector CT
Hamada et al., Japan [35]	CUA	Markov model	Third-party payer	Lifetime	3%	Patients with a diagnosed asymptomatic IPMN	MRI, CT and/or EUS: continued vs. discontinued
Huang et al., USA [36]	CUA	Markov model	Societal	Lifetime	3%	Patients with a branch duct IPMN	Surveillance vs. Prompt surgery vs. Surgery after symptoms
Lai et al., Australia [42]	CUA	Markov model	NAv	30 years	2%	Patients with a diagnosed branch duct IPMN	EUS + MRI surveillance vs. no surveillance
Lobo et al., USA [43]	CEA	DT model	NAv	15 years	NAv	Patients with asymptomatic pancreatic cysts	Consensus vs. AGA guidelines (using EUS + MRI)
Morelli et al., Italy [44]	Cost analysis	Trial-based evaluation	NAv	NAv	NAv	Patients with pancreatic cystic neoplasm	Abdominal US + MRI vs. MRI alone
Pozzi-Mucelli et al., Sweden [46]	Cost analysis	Trial-based evaluation	NAv	NAv	NAv	Patients with pancreatic cystic neoplasm	Short-protocol MRI vs. Comprehensive-protocol MRI
Richter et al., USA [47]	Cost analysis	DT model	NAv	NAv	NAv	Patients with suspected PC (as pain or weight loss)	Current practice (US, CT or clinical workup) vs. initial CA19-9
Sharib et al., USA [51]	CUA	Markov model	NAv	Lifetime	3%	Patients with a pancreatic cyst neoplasm	Upfront surgery vs. Do nothing vs. Surveillance and resection

CEA: Cost-Effectiveness Analysis, CEUS: Contrast-enhanced ultrasound, CMA: Cost-minimization analysis, CT: Computerized tomography, CUA: Cost-Utility Analysis, DT: Decision-tree, ERCP: Endoscopic retrograde cholangiopancreatography, EUS: Endoscopic ultrasound, FAMM: Familial atypical melanoma syndrome, FNA: Fine-needle aspiration, FPC: Familial pancreatic cancer, HP: Hereditary pancreatitis, HRI: high-risk individuals, ICER: Incremental cost-effectiveness ratio, IPMN: Intraductal papillary mucinous neoplasm, LY: life-year, MRCP: Magnetic resonance cholangiopancreatography, MRI: Magnetic resonance imaging, NAv: Not available, NOD: New-onset diabetes, PC: Pancreatic cancer, PET: Positron emission tomography, PJS: Peutz-Jehgers syndrome, QALY: Quality-adjusted life-year, US: Ultrasonograph

### Study populations

In all publications (*n* = 31), target populations were those with an elevated risk of pancreatic cancer. About half of all studies evaluated screening strategies targeting patients with conditions that are known to increase the risk of developing pancreatic cancer (*n* = 15; 48%). Among these screening strategies, most were model-based evaluations working with hypothetical patients (*n* = 12/15; 80%). Few studies used patient sample data (*n* = 3/15; 20%). The risk groups that were subject to screening most frequently were patients with familial pancreatic cancer (*n* = 9/15; 60%), new-onset diabetes (*n* = 6/15; 40%), different forms of genetic mutations (*n* = 5/15; 33%), and hereditary pancreatitis (*n* = 5/15; 33%).

The other studies evaluated surveillance strategies targeting patients with pre-cancerous findings or other early signs of concern (*n* = 16; 52%). Most of those studies applied a model-based design (*n* = 11/16; 69%) while other studies used patient sample data (*n* = 5/16; 31%). Patient groups that were under surveillance for pancreatic cancer predominantly had a known and asymptomatic pancreatic cyst (*n* = 13/16; 81%). Some studies evaluated surveillance in patients with pancreatic cancer-related symptoms or other indications for admission to a hospital (*n* = 3/16; 19%).

### Early detection strategies

#### Comparators

The screening studies predominantly compared screening to no screening (i.e. do nothing) as the status quo (*n* = 13/15; 87%). Surveillance strategies were compared to a do nothing option (*n* = 6/16; 38%) or against other surveillance strategies (*n* = 10/16; 63%).

#### Technologies

EUS was the most frequently evaluated technology in screening patients (*n* = 11/15; 73%), followed by MRI (*n* = 7/15; 47%), CT (*n* = 3/15; 20%), and biopsy-based tests (*n* = 5/15; 33%) (Table 2). Most studies focused on two alternatives for comparison (*n* = 9/15; 60%), but some studies evaluated more than two alternatives (*n* = 5/15; 33%). The frequency of screening was either testing patients singularly (*n* = 7/15; 47%), biannually (*n* = 1/15; 7%), annually (*n* = 7/15; 47%), or in longer intervals (*n* = 2/15; 13%).

**Table 2:** Summary of input parameters

Author(s), year, country	Start age	Testing frequency	Pancreatic cancer type/risk	Cost unit	CT cost (Sen/Spec)	EUS cost (Sen/Spec)	MRI cost (Sen/Spec)	Other costs	Sensitivity analysis
**Screening**									
Bruenderman and Martin, USA [27]	30 to 50 years	Biannual	PC: relative risk (8 to 132x)	2013 US$	325.60	601.23^1^	587.92	MRCP	No
Corral et al., USA [30]	40 years	Annual or triennial	PDAC: relative risk (> 5x)	2017 US$	-	1,525^1^ (90/90)	700^2^ (80/80)	Surgery, cancer care, death, diabetes	Deterministic Probabilistic
Draus et al., Sweden [12]	40 to 79 years	Annual	PC	2017 SEK	3,090	-	3,090	Indirect, diagnostics, surgery, cancer care	Deterministic
Ghatnekar et al., Sweden [34]	72 years	Singular	PC: incidence (0.71%)	2011 €	-	-	-	Indirect, diagnostics, cancer care	Deterministic Probabilistic
Ibrahim et al., The Netherlands [37]	45 years	Annual	PDAC	2022 €	177	773 +biopsy	363	Surgery, cancer care	Deterministic
Joergensen et al., Denmark [38]	51 years (mean)	Annual	PDAC	2015 US$	310	680	530^2^	EUS-FNA, US	*NAv*
Kowada, Japan [39]	50 years	Singular	PC: incidence (0.012 to 0.38%)	2018 US$	269 (90/87)	130.4 (91/86)	285.3 (93/89)	US, PET, cancer care	Deterministic Probabilistic
Kowada, Japan [40]	40/50/60/70 years	Annual	PC: relative risk (1.94 to 18.8x)	2020 US$	288.8 (90/87)	466.7 (91/86)	306.3 (93/89)	microRNA, CA19-9, US, PET, cancer care	Deterministic Probabilistic
Kumar et al., USA [41]	55 years	Singular	PDAC: lifetime probability (9.6%)	2018 US$	-	984.88	-	Cancer care	Deterministic Probabilistic
Peters et al., USA [45]	40 to 70 years	Singular, annual, biennial	PDAC: relative risk (2.33 to 28)	2019 US$	-	-	551	Diagnostics, surgery, cancer care	Deterministic
Rubenstein et al., USA [48]	45 years	Annual	PDAC: lifetime incidence (1.25%)	2005 US$	-	766 (72/60)	-	EUS + FNA, surgery, cancer care, diabetes	Deterministic
Rulyak et al., USA [49]	50 years	Singular	PC: prevalence (20%)	2000 US$	-	596 (90/*NAv*)	-	ERCP, surgery, cancer care, diabetes	Deterministic
Schwartz et al., USA [50]	72 years	Singular	PC: prob. in first 3 years (0.82%)	2020 US$	203	277 +biopsy	-	Cancer care, diabetes	Deterministic Probabilistic
Wang et al., USA [52]	66 years	Singular	PDAC	2020 US$	-	-	613	EUS-FNA, cancer care, diabetes, side effects	Deterministic Probabilistic
Zubarik et al., USA [53]	59 years (mean)	*NAv*	PC	2010 US$	-	248.28	-	CA19-9, EUS-FNA	*NAv*
**Surveillance**									
Alvarez et al., USA [24]	64 years (mean)	*NAv*	Pancreatic adenocarclnoma	1992 US$	495	-	623	Other modalities	*NAv*
Aronsson et al., Sweden [25]	65 years	Biannual or annual	PC: annual risk (5%)	2017 €	321	1246	406	Surgery, cancer care, diabetes	Deterministic
Bosch et al., Spain [26]	71 years (mean)	Singular	Pancreatic adenocarclnoma	2018 €	-	-	-	Diagnostics	*NAv*
Chen et al., USA [28]	*NAv*	Singular	PC: baseline prevalence (97%)	2003 US$	371.86	-	-	EUS-FNA, ERCP, cancer care, side effects	Scenario analysis
Chen et al., Canada [29]	70 years (mean)	Singular	Pancreatic lesion	2019 US$	-	719–764 (93/100)	-	-	*NAv*
Das et al., USA [31]	*NAv*	Annual or triennial	Pancreatic cystic neoplasms	2012 US$	1,000	-	1,000	EUS-FNA, surgery, cancer care	Deterministic Probabilistic
Faccioli et al., Italy [32]	60 years	Annual, 3 or 4 times/year, biannual	Pancreatic cystic neoplasms	2006 €	106.23 (69/83)	739 (83/95)	219.61 (82/96)	US	Deterministic Probabilistic
Ghaneh et al., UK [33]	66 years (mean)	*NAv*	PC	2013 £	86	-	-	PET/CT, resource use	Probabilistic
Hamada et al., Japan [35]	60 years	Biannual, annual or biennial	IPMNs	2022 US$	400	700	450	Surgery, chemotherapy	Deterministic Probabilistic
Huang et al., USA [36]	60 years	Biannual	IPMNs	2008 US$	-	-	544	Surgery, cancer care, diabetes, side effects	Deterministic Probabilistic
Lai et al., Australia [42]	65 years	Annual	IPMNs	2017 aud$	-	2,000	1,000	MRI, EUS, surgery	Deterministic Probabilistic
Lobo et al., USA [43]	55 years	*NAv*	Pancreatic cysts	2012 US$	-	1,500	1,200	Surgery	Deterministic Probabilistic
Morelli et al., Italy [44]	67 years (mean)	Biennial	Pancreatic cystic neoplasms	2017 €	-	-	480	US	*NAv*
Pozzi-Mucelli et al., Sweden [46]	64 years (mean)	*NAv*	Pancreatic cystic neoplasms	2005 €	-	-	-	MRI protocols	*NAv*
Richter et al., USA [47]	*NAv*	Singular	PC: prevalence (2 to 30%)	1987 US$	562 (80/95)	-	-	CA19-9, US, ERCP and other modalities	Scenario analysis
Sharib et al., USA [51]	60 years		Pancreatic cystic neoplasms	2018 US$	-	-	3,471	Surgery, cancer care, diabetes, side effects	Deterministic Probabilistic

^1^Includes the costs of anesthesia. ^2^Includes the costs of MRCP. CEA: Carcinoembryonic antigen, CT: Computerized tomography, EUS: Endoscopic ultrasound, ERCP: Endoscopic retrograde cholangiopancreatography, FNA: Fine-needle aspiration, IPMN: Intraductal papillary mucinous neoplasm, MDCT: Multidetector computed-tomography, MRI: Magnetic resonance imaging, NAv: not available, PC: Pancreatic cancer, PDAC: Pancreatic ductal adenocarcinoma, PET: Positron emission tomography, US: Ultrasonography

For the surveillance of patients with pancreatic precursor lesions, MRI was most frequently used (*n* = 9/16; 56%), followed by CT (*n* = 7/16; 44%), EUS (*n* = 6/16; 38%), and biopsy-based tests (*n* = 2/16; 13%). Half of all surveillance evaluations compared more than two strategies (*n* = 8/16; 50%) and two surveillance strategies were evaluated in the remaining eight studies. Patients were tested either singularly (*n* = 4/16; 25%), biannually (*n* = 4/16; 25%), annually (*n* = 5/16; 31%), or in longer intervals (*n* = 3/16; 19%). Sensitivity and specificity were reported as test performance indicators in 7 studies (*n* = 7/31; 19%).

#### Diagnosis categories

Across all studies, the diagnoses of interest were most frequently classified as “pancreatic cancer” (*n* = 12; 39%) or “pancreatic ductal adenocarcinoma” (*n* = 9; 29%), as which about 90% of pancreatic cancer diagnoses in clinical practice are classified [54]. Remaining classifications were used in surveillance evaluations and included “pancreatic cysts”, “intraductal papillary mucinous neoplasms”, and “solid pancreatic lesions”. The likelihood of developing pancreatic cancer or its precursors was incorporated via different parameters such as relative cancer risk, lifetime cancer incidence, cancer prevalence, cycle probability, or annual risk.

#### Age groups

The age at which patients were tested for pancreatic cancer from all included studies that worked with patient sample data (*n* = 9; 29%) ranged from 51 years [38] to 71 years [26]. Some model-based studies (*n* = 22; 71%) assumed base-case patients at different ages, such as 40 to 70 years [40, 45], 30 to 50 years [27], or 40 to 79 years [12]. The remaining studies assumed one age of base-case patients, so early detection strategies started within 40 (*n* = 3/22; 14%), 50 (*n* = 4/22; 18%), 60 (*n* = 7/22; 32%), or 70 years (*n* = 2/22; 9%).

### Costs

Relevant cost categories comprised inputs costs for CT, MRI, and EUS as commonly used imaging modalities. Additionally, costs of other modalities, diagnostics, surgery, cancer care, diabetes and complications were extracted, if present. About one quarter of all studies reported input costs for CT, MRI, and EUS (*n* = 8/31; 26%). In all but one of these studies [39], CT came at the lowest cost and EUS was the most costly procedure, but was often combined with additional work-up such as anaesthesia or biopsy. Seven studies reported input costs for two out of the three modalities (*n* = 7/31; 19%). Three studies listed no input costs for any of the commonly used imaging modalities (*n* = 3/31; 9%). Two studies included indirect costs by applying a societal cost perspective [12, 34]. Surgery as one potential consequence of a positive test for pancreatic cancer was included by 13 studies (*n* = 13/31; 42%). Another 17 studies included general costs of pancreatic cancer care episodes (*n* = 17/31; 55%). Complication-related costs were reported by 4 studies (*n* = 4/31; 13%).

### Utilities

The majority of studies included utility values as outcome measure (*n* = 20/31; 65%) (Appendix 3). In six studies, QALYs were measured by using the EQ-5D as a generic instrument [12, 25, 33, 34, 37, 39] and one study relied on expert opinion [35]. In most studies, the instrument to measure patient utilities could not be determined (*n* = 13/20; 65%). Detrimental effects of undergoing testing procedures (e.g., invasive testing procedures) on quality of life was included in five studies [30, 32, 41, 43, 50]. The majority of cost-utility analyses (13/20; 65%) displayed sensitivity ranges around the utility inputs. Out of these, two studies used beta distributions [35, 50] and one used a normal distribution [41]. Four studies used age-adjusted utilities [25, 32, 33, 36] and one study adjusted utilities to the country context [34].

### Study outcomes and uncertainty assessment

#### Full economic evaluations

The majority of economic evaluations reported an incremental cost-utility ratio (ICUR) as main outcome and compared it to a given willingness-to-pay threshold (*n* = 20; 65%) (Table 3). Some studies calculated a range of ICURs given different scenarios (*n* = 6/20; 30%). Three studies reported costs both per incremental QALY gained and per life-year saved [25, 38, 50]. One study exclusively reported a ratio of costs per saved life-year [49]. Other outcomes relating costs to effects were cost per prevented pancreatic cancer death [37] or per pancreatic cancer diagnosis [28, 43, 53]. Secondary economic outcomes included costs per detection procedure [29, 46, 47], or per patient [24, 26, 44], which reflected provider-relevant costs of early detection.

**Table 3:** Summary of study results and conclusions

Author(s), year, country	Main study outcomes	ICUR (cost per QALY gained)	Other output	Willingness-to-pay per QALY	CE?
**Screening**					
Bruenderman and Martin, USA [27]	Total costs; average survival after diagnosis; average age of PC death; LYs added	-	$/added LY: 356.42 to 1,141.77	-	-
Corral et al., USA [30]	Total costs per QALY gained	Screening dominant to $84,020	-	$0 to $100,000	Partly
Draus et al., Sweden [12]	Total costs per QALY gained; false positive cases and their extra costs	-17,829 € to 2,000,000 €	-	100,000 €	Partly
Ghatnekar et al., Sweden [34]	Total costs per QALY gained; LYs gained	13,466 €	-	56,000 €	Yes
Ibrahim et al., The Netherlands [37]	Total costs per QALY gained; PC detection number, mean life expectancy, mortality	14,000 €	Lifetime PC risk needs to be 10% for CE	50,000 €	Yes
Joergensen et al., Denmark [38]	Total costs per QALY and LY gained; PC detection	$47,867 to $58,647	$/LY saved: 35,493 to 47,156	$50,000	Partly
Kowada, Japan [39]	Total costs per QALY gained	Screening dominant to $214,488	-	$50,000	Partly
Kowada, Japan [40]	Total costs per QALY and LY gained; life expectancy LYs, PC deaths	Range of ICURs; probabilistic results	-	$100,000	Partly
Kumar et al., USA [41]	Total costs per QALY gained; required screening parameters for CE	$82,669	-	$100,000	Partly
Peters et al., USA [45]	Total costs per QALY gained	$29,000 to $48,000,000	-	$100,000 and $200,000	Partly
Rubenstein et al., USA [48]	Total costs per QALY gained; mortality, quality of life, operative complications, costs	Do nothing dominant	-	*NAv*	No
Rulyak et al., USA [49]	Total costs per LY gained	-	$/LY saved: 16,885	*NAv*	-
Schwartz et al., USA [50]	Total costs per QALY and LY gained	$65,076	$/LY saved: 53,421	$50,000 and $100,000	Partly
Wang et al., USA [52]	Total costs per QALY gained; PC minimum risk for screening to be cost-effective	$63,045 and $116,911	Screening is CE when PDAC risk > 1–2%	$100,000 and $150,000	Yes
Zubarik et al., USA [53]	Total costs per detected case, number detected	-	Detection cost/PC: $41,133, /neoplasia: $8431	-	-
**Surveillance**					
Alvarez et al., USA [24]	Test performance, costs to diagnose pancreatic cancer	-	Testing algorithm would save $485 per patient	-	-
Aronsson et al., Sweden [25]	Total costs per LY and QALY gained	31,682 €	$/LY saved: 15,412 €	100,000 €	Yes
Bosch et al., Spain [26]	Diagnostic effectiveness and associated costs	-	€347.76/ quick-diagnosis unit patient and €634.36/ inpatient	-	-
Chen et al., USA [28]	Total costs per diagnosis	-	EUS-FNA dominant by costs per diagnosis	-	-
Chen et al., Canada [29]	Diagnostic accuracy/characteristics, resource use, cost-minimization data	-	EUS-FNA + ROSE ($719) vs. EUS-FNB ($764)	-	-
Das et al., USA [31]	Total costs per QALY gained; number needed to treat	$62 for integrated mutational profiling	-	$20,000 to $100,000	Yes
Faccioli et al., Italy [32]	Total costs per QALY gained	-	Ultrasound dominated guidelines	30,000€	-
Ghaneh et al., UK [33]	Total costs per QALY gained	£19,445 to £53,557 for PET/CT	-	£20,000 and £30,000	Partly
Hamada et al., Japan [35]	Total costs per QALY gained; optimal age to stop surveillance, cancer-related deaths	$39,761 to $139,088	Opt. age to stop surveillance: 76–78 (male), 70–84 (female)	$100,000	Partly
Huang et al., USA [36]	Total costs per QALY gained	$20,096	-	$50,000	Partly
Lai et al., Australia [42]	Total costs per QALY gained	$34,758 and $64,555	-	$50,000	Partly
Lobo et al., USA [43]	Total costs per additionally detected PC, PC deaths	-	Additional cancer detected by consensus guideline: $3.6 mio.	-	-
Morelli et al., Italy [44]	Mean cost of surveillance per patient	-	Cost per patient: 366.4€	-	-
Pozzi-Mucelli et al., Sweden [46]	Cost reduction from using a shorter surveillance protocol	-	Short-protocol was 25% of comprehensive-protocol cost	-	-
Richter et al., USA [47]	Total costs per patient	-	CA19-9: $848 to $1413, US: $1186 to $1848	-	-
Sharib et al., USA [51]	Total costs per QALY gained; required test parameters	$171,143 and $80,707	Diagnostic specificity must be > 67% to be cost-effective	$100,000	Partly

CE: cost-effective, CT: Computed tomography, EUS-FNA: Endoscopic-ultrasound fine-needle aspiration, ICUR: Incremental cost-utility ratio, PC: Pancreatic cancer, PET: Positron emission tomography, QALY: Quality-adjusted life-year, WTP: Willingness-to-pay

Among all 20 cost-utility analyses, three studies found screening to be the dominant strategy compared to no screening in selected base-case scenarios [30, 39, 40]. On the opposite, one study reported no screening as the dominant strategy [48]. Six publications calculated the ICUR as a point estimate and concluded cost-effectiveness of early detection based on a willingness-to-pay threshold [25, 31, 32, 34, 37, 52]. The remaining 13 studies reported partly cost-effective results due to changing results in sensitivity analyses or to different cost-effectiveness thresholds [12, 30, 33, 35, 36, 38–42, 45, 50, 51]. Deterministic and probabilistic sensitivity analysis were conducted by 13 studies, six studies conducted only deterministic [25, 28, 37, 45, 48, 49] and one study conducted only probabilistic sensitivity analysis [33].

Other findings reported in full economic evaluations included the optimal age to stop surveillance of intraductal papillary mucinous neoplasms, which was found to be 76–78 (male) and 70–84 (female) depending on the pancreatic lesion size [35]. Another study concluded that for high-risk cysts a minimum specificity of 67% is required for surveillance to be cost-effective [51].

#### Partial economic evaluations

Out of nine cost analyses, two studies evaluated screening procedures. A model-based cost comparison found MRI-based screening in high-risk individuals to be affordable for the US health care system in 2016 [27]. Another study calculated the costs of detecting one case of pancreatic cancer or a precursor case via screening high-risk individuals, but did not provide further interpretation of these results [53]. Seven studies compared costs of surveillance. Four studies compared detection technologies reporting comparable costs of two EUS methods [29], lower costs in favour of a short-MRI-protocol compared to a long protocol [46], cost savings in an ambulatory compared to inpatient setting [26], and cost savings due to inclusion of ultrasound complementary to MRI [44]. Three studies calculated hypothetical cost savings in surveillance either suggesting a diagnostic algorithm based on evaluated test performance [24], or using EUS-FNA as the most cost saving modality [28], or using CA19-9 radioimmunoassay as the most cost saving technology for testing respective patients [47].

### Cost effectiveness assessment

#### Cost-effectiveness of screening

Four studies compared screening strategies against each other in homogeneous risk-cohorts such familial pancreatic cancer [39, 48] or diabetes patients [40, 50, 54]. Rubenstein et al. found “do-nothing” to be the dominant strategy compared to annual screening in a target population with a pancreatic cancer lifetime incidence of 1.25% [48]. On the contrary, a study from Japan found screening to be cost-effective in familial cancer patients at higher relative risks and depending on the modality used [39]. Wang et al. concluded screening of new-onset diabetes patients to be cost-effective, if the cancer risk was sufficiently high (> 1–2%) and assuming different willingness-to-pay thresholds [54]. Another Japanese study reported a broad range of ICURs in stratified diabetes patient populations and concluded cost-effectiveness for screening high-risk patients using micro-RNA [40]. Schwartz et al. assessed the cost-effectiveness of screening new-onset diabetes patients via CT finding the strategy to be likely cost-effective, but suggested, that at least a quarter of detected patients needed to be resectable for these results to hold [50].

Other studies compared two (screening versus no screening) [41, 45] or three strategies (additional screening strategy) across various high-risk groups [30]. Corral et al. simulated screening of different target populations at different intervals comparing either MRI, EUS, or no screening modality [30]. The study suggests MRI screening to be cost-effective in populations at moderate risk and EUS in populations at high risk [30]. In another study, EUS-based screening was partially cost-effective and the key driving factors were found to be test parameters, cancer probability and life expectancy after resection [41]. The only included microsimulation model showed MRI and EUS combined may be cost-effective for screening highest-risk groups, but in order to reach such conclusions in moderate risk patients higher willingness-to-pay thresholds may be necessary as well as less screens per patient [45].

Two studies from Sweden assessed the hypothetical performance of a biomarker test versus no screening in high-risk populations and concluded that such a test was either cost-effective [34] or partly cost-effective depending on test parameters and individuals to be screened [12]. Additionally, the latter study suggested that even a test with nearly perfect sensitivity and specificity would not justify screening the general population given high excess costs of false-positive cases [12].

By evaluating cost-effectiveness of pancreatic cancer screening in real patient cohorts, two studies found the respective programs to be at least partly cost effective [37, 38]. In the Netherlands, 347 gene mutation carriers were tested annually via MRI, which resulted in 31 detected and 22 resected cases. However, the authors noted that the cancer risk needs to be at least 10% to constitute cost-effectiveness in other settings [37]. In a Danish cohort, 71 patients with different familial predispositions were tested annually via EUS with their results suggesting cost-effectiveness particular in the familial pancreatic cancer group [38].

#### Cost-effectiveness of surveillance

Four Markov modelling studies compared surveillance to prompt surgery and to a “do-nothing” option in 60 or 65 year old patients that had precancerous lesions [25, 31, 36, 51]. Two studies found the surveillance strategy to be cost-effective [25, 31] and two reported partial cost-effectiveness [36, 51]. A Swedish study suggested that initial surveillance was cost-effective in managing low-risk pancreatic lesions despite better effectiveness of the immediate surgery strategy [25]. Huang et al. also found surgery to be most effective but surveillance to provide the best cost-effectiveness ratio and 88.1% of patients to be in a $50,000 per QALY margin. According to Sharib et al. diagnostic specificity had to be > 67% for risk stratification for high-risk cysts in order to be cost-effective, which was not the case for low-grade cysts [51].

Two Markov models simulated patients with intraductal papillary mucinous neoplasms as pancreatic precursor lesions and the cost-effectiveness of MRI- and EUS-based surveillance strategies [35, 42]. Both reported partial cost-effectiveness. Hamada et al. reported sex, lesion types, and comorbidity levels as determinants of surveillance discontinuation, which points to more personalized surveillance approaches also with regard to cost-effectiveness [35].

Finally, two economic evaluations focused on specific diagnostic technologies, namely positron emission tomography combined with CT [33] or contrast-enhanced ultrasound [32] for patients with known pancreatic cysts. Ghaneh et al. compared adding the former technology to the standard diagnostic work-up and found partially cost-effective results based on 550 patients enrolled in the study [33]. The inclusion of contrast-enhanced ultrasound in cyst surveillance was found to be cost-effective, but only the direct cost of imaging were included [32].

## Discussion

Out of 22 cost-effectiveness analyses, six reported cost-effectiveness underneath a favourable willingness-to-pay threshold [25, 31, 32, 34, 37, 52], one concluded no screening was the dominant strategy [48], and 13 evaluations reported mixed results, so early detection was cost-effective in some scenarios [12, 30, 33, 35, 36, 38–42, 45, 50, 51]. These results show that early detection strategies for pancreatic cancer could be cost-effective in certain target populations. Furthermore, results from partial economic evaluations point to the potential of saving costs by using less resource demanding early detection strategies. The identification of cost-effective early detection strategies could therefore contribute to improving the overall quality of pancreatic cancer care as argued by the US Institute of Medicine [55]. However, it is important to read the results with caution due to high methodological heterogeneity. Furthermore, this systematic review is aimed at an initial collection of economic evidence in the area of pancreatic cancer early detection, which means that no systematic statement can yet be made about the cost-effectiveness of specific strategies.

In comparison with the most recent systematic review on pancreatic cancer which included economic evaluations until 2015 [18], this review has identified substantially more evaluations of screening and surveillance strategies (*n* = 31) than the previous study (*n* = 6). The higher number of included studies before 2015 in this study was likely due to different search strategies used. The ratio of full to partial economic evaluations at about two thirds to one third was similar. The previous review assessed study quality based on the 10-item Drummond checklist rating studies individually reporting better quality in full than in partial economic evaluations. This review found a similar trend. The use of quality assessment tools for both full and partial economic evaluations thus rather works in favour of full economic evaluations, so the development of hybrid tools may be of help for future reviews.

The included literature shows that compared to the period before 2015 [24, 28, 34, 36, 47–49, 53], the number of publications on the topic has nearly tripled since then [12, 25–27, 29–33, 35, 37–46, 50–52]. Those to be tested for pancreatic cancer had risk predispositions, which could be divided into conditions associated with a higher pancreatic cancer risk as well as known pre-cancerous findings or symptoms. CT, MRI and EUS were identified as the most common imaging modalities for pancreatic cancer detection in line with previous literature [56]. In a comparison of input costs for the three modalities, CT was least costly followed by MRI and EUS had the highest costs with the exception of one study [39]. In view of another publication according to which MRI is the most expensive procedure, this result raises questions as to which cost components are included in an EUS or MRI [57]. For example, two studies included anaesthesia costs [27, 30] and two other studies included the cost of biopsy as part of EUS [37, 50]. In the case of MRI, two studies included the costs of an MRCP [30, 38]. Given that the hierarchy of input costs can have a decisive influence on output costs, it will be important in future studies to define the individual cost components of a procedure. This will also facilitate meta-analytical analyses, as early detection strategies will be more comparable across studies.

There is considerable diversity in screening strategies, which also explains the variation in results. The studies can broadly be categorised into screening and surveillance studies based on the patient populations. In line with an early definition of screening [58], all screening studies met the definition of selective screening, which describes only testing high-risk patients. Surveillance examines patients with some form of complaint on an individual level, which was fulfilled by all studies evaluating surveillance strategies [58]. A further categorisation of the studies was possible with regard to model-based and trial-based studies. Trial-based studies were the minority [24, 26, 29, 33, 37, 38, 44, 46, 53], which can be explained by the difficulty of following patient cohorts over a longer time period. In addition, the risk of pancreatic cancer in the general population is relatively low, therefore large or selected high-risk cohorts will be needed to study early detection cases. Thus, the conclusions regarding cost-effectiveness that can be drawn are mainly based on model-based economic evaluations. Health economic models aim to approximate reality as closely as possible to produce reliable results.

As demonstrated in the reporting completeness assessment, some reporting items that are required in economic evaluations were incomplete. The bias assessment showed that only few studies took a societal perspective [12, 34, 36, 49] and alternative perspectives may have neglected relevant costs. In addition, not all evaluations considered a lifetime horizon, which may have resulted in a truncated view. Another area for methodological improvement would be the discounting of future costs and effects, which was only done in 60% of the studies. In addition to these methodological aspects, a clear patients of interest definition is required for the evaluation of early cancer detection strategies. In three studies it was not possible to determine how old the tested patients were [28, 31, 47] and in two studies the test frequency was unclear [43, 51]. Future economic evaluations should define early detection strategies as precisely as possible in terms of target group and technological aspects in order to achieve better replicability. Finally, all but one study applied cohort-based models to the respective decision problems. Peters et al. used a microsimulation model to simulate the natural history of pancreatic cancer, which allows individual level modelling of patients and thus more complex simulations [45]. The use of such modelling approaches may be supported by another study suggesting more tailored approaches to screening initiation and discontinuation [35].

Some studies demonstrated the challenges of clearly defining eligible patients for pancreatic cancer surveillance. The referral of mostly symptomatic patients – around half of the patients presented with weight loss and abdominal pain – to diagnostics may disqualify to be classified as early detection [26]. Nevertheless, an explanation for referring patients to the hospital, which may have been due to precancerous findings, was missing. In another study, the criteria for referring patients to be tested were also not stated, so at least part of the patient population was assumed to fall under early detection [24]. These exceptions illustrate that in pancreatic cancer, unlike other diseases, symptom-motivated surveillance of patients may no longer fall under early detection, since the cancer may have progressed too far. The aforementioned definition of patients with a complaint therefore usually means an incidental finding of precancerous lesions in patients, which is then observed individually over a longer period. Nor can the general definition of screening be applied to pancreatic cancer in the same way; until now, patient groups had to be pre-selected in order to undergo screening procedures.

In order to facilitate the comparability of economic evaluations in pancreatic cancer early detection, we recommend that future studies adhere to the CHEERS checklist [22], with special attention to the following focus areas in the evaluation of pancreatic cancer early detection: The increased risk of pancreatic cancer in the target population compared to the general population should be explicitly stated. Early detection interventions should be compared against the standard of care. Input costs should reflect the resources required for the screening algorithm as well as follow-up services described in guidelines, e.g. for further diagnostics and treatment, ideally determined and assigned bottom-up using real-world evidence. Utility values should be incorporated transparently, i.e. by naming the instrument used and stating the measurement uncertainty for assessing health states. Outcomes should be assessed over a lifetime horizon and the choice of a discount rate should be well justified.The contributions of this study should be understood in the context of its limitations. It must be taken into account that this systematic review may have missed economic evaluations that were not published or that were published in another language than English. In particular, the rather favourable results of many economic evaluations highlight the chance of publication bias, so publications with positive results are more likely to be published than those with negative results. Furthermore, despite the effort of performing reporting quality and risk of bias assessment independently, this process may have been affected by subjective judgements of the two reviewers. The application of both checklists to a study sample consisting of both full and partial economic evaluations may have not been an ideal fit to assess partial economic evaluations. Finally, the description of study results was done following a qualitative approach, which may have introduced bias by highlighting certain findings. However, all studies were furthermore presented in tabular form to provide a structured overview as well. The heterogeneity in the reviewed literature with regard to study designs and outcomes did not allow the use of statistical or meta-analytic techniques to present pooled results.

## Conclusion

This study has reviewed a substantial body of literature on economic aspects of pancreatic cancer early detection and demonstrated a growing interest in this aspect of cancer care. Comparisons of early detection strategies against the standard of care or alternative strategies have shown promising results regarding its cost-effectiveness. However, a large variety in early detection approaches does not allow any endorsements of any specific testing strategies yet. Moreover, pancreatic cancer early detection strategies should be classified as either screening or surveillance based on the patients to be tested. Future economic evaluations of pancreatic cancer early detection should adhere to a clear definition of patients to be tested based on this classification. In both cases, pancreatic cancer early detection requires the selection of patient cohorts with an elevated risk of the cancer. This is because of currently limited modalities that would cause a high psychological and economic burden due to an unacceptable number of false-positive patients. From a methodological standpoint, the use of microsimulation modelling in one study is a promising development in making health economic modelling of pancreatic cancer screening more advanced. Due to its low prevalence and the long time horizons of screening, pancreatic cancer early detection will likely rely on model-based evaluations in the future. In order to guide health economic decisions on early detection strategies, this field will require further research effort to identify optimal testing sequences, treatment options and diagnostic tools.

## Electronic supplementary material

Below is the link to the electronic supplementary material.

Supplementary Material 1

## Data Availability

Data sharing is not applicable to this article as no datasets were generated or analysed during the current study.
