# Supplementary material for: Economic evaluations of early detection strategies for pancreatic cancer: a systematic review
Source: Eur J Health Econ. 2025 Jun 5;26(9):1655–70. doi: 10.1007/s10198-025-01793-4 (PMC12618292; doi:10.1007/s10198-025-01793-4)
Supplement: Supplementary file 1 — Supplementary Material 1 [file 10198_2025_1793_MOESM1_ESM.docx]

**Supplementary information**

Economic evaluations of early detection strategies for pancreatic cancer: A systematic review

The European Journal of Health Economics

Corresponding author: Robert Wittram, University Medical Center Hamburg-Eppendorf, Department of Health Economics and Health Services Research, [r.wittram@uke.de](mailto:r.wittram@uke.de)

All authors: Robert Wittram, Léon Kreis, Hans-Helmut König, Christian Brettschneider

**Appendix 1.** Database Search Method

**PubMed:**

(#1) "Economics"[Mesh:NoExp] OR "Costs and Cost Analysis"[mh] OR "Economics, Nursing"[mh] OR "Economics, Medical"[mh] OR "Economics, Pharmaceutical"[mh] OR "Economics, Hospital"[mh] OR "Economics, Dental"[mh] OR "Fees and Charges"[mh] OR "Budgets"[mh] OR budget*[Tiab] OR economic*[Tiab] OR cost[Tiab] OR costs[Tiab] OR costly[Tiab] OR costing[Tiab] OR price[Tiab] OR prices[Tiab] OR pricing[Tiab] OR pharmacoeconomic*[Tiab] OR "pharmaco-economic*"[Tiab] OR expenditure[Tiab] OR expenditures[Tiab] OR expense[Tiab] OR expenses[Tiab] OR financial[Tiab] OR finance[Tiab] OR finances[Tiab] OR financed[Tiab] OR "value for money"[Tiab] OR "monetary value*"[Tiab] OR "models, economic"[mh] OR "economic model*"[Tiab] OR "markov chains"[mh] OR markov[Tiab] OR "monte carlo method"[mh] OR "monte carlo"[Tiab] OR "Decision Theory"[mh] OR "decision tree*"[Tiab] OR "decision analy*"[Tiab] OR "decision model*"[Tiab]

(#2) (("pancreatic neoplasms"[Mh] OR ("pancreatic"[Tiab] AND "neoplasms"[Tiab]) OR "pancreatic neoplasms"[Tiab]) OR ("pancreatic neoplasms"[Mh] OR ("pancreatic"[Tiab] AND "neoplasms"[Tiab]) OR "pancreatic neoplasms"[Tiab] OR ("pancreatic"[Tiab] AND "cancer"[Tiab]) OR "pancreatic cancer"[Tiab]) OR ("pancreatic neoplasms"[Mh] OR ("pancreatic"[Tiab] AND "neoplasms"[Tiab]) OR "pancreatic neoplasms"[Tiab] OR ("pancreas"[Tiab] AND "neoplasms"[Tiab]) OR "pancreas neoplasms"[Tiab]) OR ("pancreatic neoplasms"[Mh] OR ("pancreatic"[Tiab] AND "neoplasms"[Tiab]) OR "pancreatic neoplasms"[Tiab] OR ("pancreas"[Tiab] AND "cancer"[Tiab]) OR "pancreas cancer"[Tiab]))

(#3) ("screening"[Tiab] OR "early detection"[Tiab] OR "diagnosis"[Tiab] OR "surveillance"[Tiab] OR “Early Detection of Cancer” [Mh])

(#4) #1 AND #2 AND #3

(#5) (“Systematic review”[Publication Type] OR “Meta-Analysis” [Publication Type] OR “letter”[Publication Type] OR “newspaper article”[Publication Type] OR “Congress”[Publication Type] OR “review” [Publication Type] OR “comment”[Publication Type])

(#6) #4 NOT #5

**Web of Science:**

Search in Web of Science: Title Or Abstract

(#1) (cost* OR economic evaluation)

(#2) (pancreatic cancer OR pancreatic neoplasm)

(#3) (screening OR early detection OR diagnosis OR surveillance)

(#4) #1 AND #2 AND #3

**Econlit:**

Search in Econlit: All Text

(cost* OR economic evaluation) AND (pancreatic cancer OR pancreatic neoplasm) AND (screening OR early detection OR diagnosis OR surveillance)

**Appendix 2.** ECOBIAS assessment results

**Appendix 3.** Overview of utility input parameters

| **Author, Year** | **Instrument** | **Screening effect*** | **Sensitivity range** | **Context**  **adjustment** | **Model driver** | **Base case** | **Localized/ Regional** | **Metastatic** |
| --- | --- | --- | --- | --- | --- | --- | --- | --- |
| Corral et al., 2019 | NAv | Yes | Yes | No | No | 1.00 | 0.9 | - |
| Draus et al., 2022 | EQ-5D | No | No | No | - | - | - | - |
| Ghatnekar et al., 2013 | EQ-5D | No | No | Country | - | - | 0.79-0.83 | 0.76 |
| Kowada, 2020 | EQ-5D | No | Yes | No | No | 1.00 | 0.79-0.83 | 0.76 |
| Kowada, 2022 | NAv | No | Yes | No | No | 0.92 | 0.80-0.83 | 0.76 |
| Kumar et al., 2021 | NAv | Yes | Normal | No | Yes | 1.00 | - | 0.50 |
| Rubenstein et al., 2007 | NAv | No | Yes | No | Yes | 1.00 | - | - |
| Schwartz et al., 2022 | NAv | Yes | Beta | No | Yes | 0.92 | 0.78-0.80 | 0.73 |
| Wang et al., 2022 | NAv | No | Yes | No | Yes | 0.82 | 0.72-0.73 | 0.72 |
| Ibrahim et al., 2023 | EQ-5D | No | No | No | Yes | 0.85 | 0.72-0.78 | - |
| Joergensen et al., 2016 | NAv | No | No | No | - | 1.00 | - | 0.50 |
| Aronsson et al. 2018 | EQ-5D | No | Yes | Age | Yes | 0.80 | - | - |
| Das et al., 2015 | NAv | No | Yes | No | No | 1.00 | 0.90 | 0.50 |
| Faccioli et al., 2022 | NAv | Yes | No | Age | - | 0.80 | - | 0.68 |
| Hamada et al., 2022 | Expert opinion | No | Beta | No | No | 1.00 | - | 0.50 |
| Huang et al., 2010 | NAv | No | Yes | Age | Yes | 1.00 | - | 0.69 |
| Lai et al., 2023 | NAv | No | No | No | - | 0.84 | - | - |
| Lobo et al., 2020 | NAv | Yes | No | No | - | 1.00 | 0.90 | 0.50 |
| Sharib et al., 2020 | NAv | No | Yes | No | Yes | 0.88 | - | 0.69 |
| Ghaneh et al., 2018 | EQ-5D | No | Yes | Age | No | - | - | - |

*Captures whether any changes in quality of life through the screening procedure were taken into account.
